# Inhibition of I **κ** B-**α** phosphorylation at serine and tyrosine acts independently on sensitization to DNA damaging agents in human glioma cells

**DOI:** 10.1054/bjoc.1999.0872

**Published:** 1999-12-08

**Authors:** J Miyakoshi, K Yagi

**Affiliations:** Department of Radiation Genetics, Graduate School of Medicine; Department of Zoology, Graduate School of Science, Kyoto University, Yoshida-Konoe-cho, Sakyo-ku, 606-8501, Japan

**Keywords:** IκB-α, NF-κB, phosphorylation, DNA damaging agents, human glioma cells

## Abstract

Molecular mechanisms and/or intrinsic factors controlling cellular radiosensitivity are not fully understood in mammalian cells. The recent studies have suggested that nuclear factor κB (NF-κB) is one of such factors. The activation and regulation of NF-κB are tightly controlled by IκB-α, a cellular inhibitory protein of NF-κB. Most importantly, phosphorylation regulates activity of the inhibitor IκB-α, which sequesters NF-κB in the cytosol. Two different pathways for the phosphorylation of IκB-α are demonstrated, such as serine (at residues 32 and 36) and tyrosine (at residue 42) phosphorylations. To assess a role of the transcription factor, NF-κB, on cellular sensitivity to DNA damaging agents, we constructed three different types of expression plasmids, i.e. S-IκB (mutations at residues 32 and 36), Y-IκB (mutation at residue 42) and SY-IκB (mutations at residues 32, 36 and 42). The cell clones expressing S-IκB and Y-IκB proteins became sensitive to X-rays as compared with the parental and vector-transfected cells. The cell clones expressing SY-IκB were further radiosensitive. By the treatment with herbimycin A, an inhibitor of phosphorylation, the X-ray sensitivity of cells expressing SY-IκB did not change, while that of the cells expressing S-IκB and Y-IκB and the parental cells was enhanced. Change in the sensitivity to adriamycin and UV in those clones was very similar to that in the X-ray sensitivity. The inhibition of IκB-α phosphorylation at serine and tyrosine acts independently on the sensitization to X-rays, adriamycin and UV. These findings suggest that the transcriptional activation induced by NF-κB may play a role in the DNA damage repair. The present study proposes a possibility that the inactivation of NF-κB by inhibition of both serine and tyrosine phosphorylations may be useful for the treatment of cancer in radio- and chemotherapies. © 2000 Cancer Research Campaign


*British Journal of Cancer* (2000) **82**(1), 28–33
© 2000 Cancer Research Campaign
Article no. bjoc.1999.0872

### Inhibition of IκB-α phosphorylation at serine and tyrosine acts independently on sensitization to DNA damaging agents in human glioma cells

**J Miyakoshi[1] and K Yagi[2]**

[1]Department of Radiation Genetics, Graduate School of Medicine and [2]Department of Zoology, Graduate School of Science, Kyoto University, Yoshida-Konoe-cho, Sakyo-ku, Kyoto 606-8501, Japan

**Summary** Molecular mechanisms and/or intrinsic factors controlling cellular radiosensitivity are not fully understood in mammalian cells. The recent studies have suggested that nuclear factor κB (NF-κB) is one of such factors. The activation and regulation of NF-κB are tightly controlled by IκB-α, a cellular inhibitory protein of NF-κB. Most importantly, phosphorylation regulates activity of the inhibitor IκB-α, which sequesters NF-κB in the cytosol. Two different pathways for the phosphorylation of IκB-α are demonstrated, such as serine (at residues 32 and 36) and tyrosine (at residue 42) phosphorylations. To assess a role of the transcription factor, NF-κB, on cellular sensitivity to DNA damaging agents, we constructed three different types of expression plasmids, i.e. S-IκB (mutations at residues 32 and 36), Y-IκB (mutation at residue 42) and SY-IκB (mutations at residues 32, 36 and 42). The cell clones expressing S-IκB and Y-IκB proteins became sensitive to X-rays as compared with the parental and vector-transfected cells. The cell clones expressing SY-IκB were further radiosensitive. By the treatment with herbimycin A, an inhibitor of phosphorylation, the X-ray sensitivity of cells expressing SY-IκB did not change, while that of the cells expressing S-IκB and Y-IκB and the parental cells was enhanced. Change in the sensitivity to adriamycin and UV in those clones was very similar to that in the X-ray sensitivity. The inhibition of IκB-α phosphorylation at serine and tyrosine acts independently on the sensitization to X-rays, adriamycin and UV. These findings suggest that the transcriptional activation induced by NF-κB may play a role in the DNA damage repair. The present study proposes a possibility that the inactivation of NF-κB by inhibition of both serine and tyrosine phosphorylations may be useful for the treatment of cancer in radio- and chemotherapies. © 2000 Cancer Research Campaign

**Keywords:** IκB-α; NF-κB; phosphorylation; DNA damaging agents; human glioma cells

Ionizing radiation activates immediately early genes, such as c-jun, c-fos and nuclear factor κB (NF-κB) (Hallahan et al, 1991; Wilson et al, 1993; Mohan and Meltz, 1994). The products of these genes are transcription factors involved in regulation of the gene expression associated with cell growth, healing of tissue injury and inflammation. Transfection with certain oncogenes has been found to cause cellular radiation resistance (Sklar, 1988; Kasid et al, 1989; Pirollo et al, 1993). These results suggest that related signalling pathways may be involved in acquisition of radiation resistance. It has been demonstrated that introduction of a truncated IκB-α corrected both hypersensitivity to ionizing radiation and the aberrant activation of NF-κB in ataxia telangectasia (AT) cells (Jung et al, 1995). Therefore, activation of transcription factor(s) may be involved in cellular radiosensitivity.

One of the proteins of the Rel/NF-κB family, NF-κB, is a transcription factor which binds specifically to κB motifs as homo- or heterodimers and regulates the expression of many genes (reviewed in Baeuerle, 1991; Verma et al, 1995). The activation and regulation of NF-κB are tightly controlled by IκB-α, a cellular inhibitory protein of NF-κB. Normally, IκB-α binds to NF-κB, thereby preventing the translocation of NF-κB into nucleus (Baeuerle and Baltimore, 1988; Beg et al, 1992). Some of the stimuli including tumour necrosis factor-α (TNF-α), ultra-violet (UV) and ionizing radiation cause the degradation and disappearance of IκB-α (Alkalay et al, 1995; Scherer et al, 1995), and then the NF-κB translocates to the nucleus.

Phosphorylation is involved in the regulation of the activity of many transcription factors. The NF-κB is a critical regulator of cytokine-inducible gene expression (Baeuerle and Henkel, 1994). Most importantly, phosphorylation regulates activity of the inhibitor IκB-α, which sequesters NF-κB in the cytosol. For the activation of NF-κB, two different pathways for the phosphorylation of IκB-α are demonstrated. One is serine (at residues 32 and 36) phosphorylation, which induces subsequent ubiquitin-dependent degradation of IκB-α (Brockman et al, 1995; Brown et al, 1995; Traenckner et al, 1995). The other is tyrosine (at residue 42) phosphorylation, which has the potential to directly couple NF-κB to surface receptor associated tyrosine kinases, but not induce the degradation of IκB-α (Imbert et al, 1996).

We constructed three different types of expression plasmids, such as S-IκB containing Ser-to-Arg (AGC-CGC) mutations at residues 32 and 36, Y-IκB containing Tyr-to-Phe (TAC-TTC) mutation at residue 42, and SY-IκB containing all these mutations. To assess the role of NF-κB activation on cellular sensitivity to X-rays, adriamycin and UV, we examined the sensitivity to these DNA damaging agents in human malignant glioma cells expressing S-IκB, Y-IκB and SY-IκB proteins.

*Received 14 January 1999*
*Revised 17 June 1999*
*Accepted 21 June 1999*

*Correspondence to:* J Miyakoshi

### MATERIALS AND METHODS

### Cell lines and culture conditions

The established human malignant glioma cell line, MO54, was used. The cells were kindly supplied from Dr RS Day, Cross Cancer Institute, Edmonton, AB, Canada. Cells were cultured in Dulbecco's modified Eagle medium plus 10% fetal bovine serum (Hyclone Laboratories, Inc., Logan, UT, USA) at 37°C with 95% air and 5% carbon dioxide.

### Mutant constructions and transfections

For S-IκB plasmid, the IκB-α cDNA (a kind gift from Dr J Inoue, The Institute of Medical Science, University of Tokyo, Japan) inserted into the pBluescript II S/K plasmid was used as a template for further polymerase chain reaction (PCR) amplification. Mutagenic primers for $^{32}$Ser to $^{32}$Arg and $^{36}$Ser to $^{36}$Arg, such as MAD-LPU; 5′-CGACCGCCACGA<u>CGC</u>CGGCCTGGA<u>CGC</u>-CATGAAAGACG-3′ and MAD-LPL; 5′-CGCTTTCATG-<u>GCG</u>TCCAGGCCG<u>GCG</u>TCGTGGCGGTCG-3′, were used in PCR reaction. The reaction mixture was as follows; 1 μl of pB-MAD3 plasmid (37 μg ml$^{-1}$), 1 μl of each primer (1 pmol μl$^{-1}$), 4 μl of 25 mM magnesium chloride, 5 μl of dNTP mix (2 mM each), 0.5 μl of KOD polymerase (5 units μl$^{-1}$), 5 μl of 10 × KOD buffer and 34 μl of distilled water. PCR condition was 15 cycles of 30 s at 98°C, 30 s at 55°C and 2 min at 74°C with a final extension at 72°C for 3 min. The mixture was treated with *Dpn*I for the degradation of the template plasmid. The plasmid sample was introduced into *Escherichia coli* JM109 strain by electroporation. After 1 h incubation with SOC medium, the cells were plated on LB dishes containing ampicillin. The S-IκB-α cDNA inserted in the pBluescript II S/K plasmid were refined by using QIAprep Spin Miniprep kit (Qiagen, Germany) and checked by digestion with *Eco*RI. Then, sequencing of the S-IκB-α cDNA was done with a Prism Dye Primer Cycle Sequencing Kit (–21M13) (Applied Biosystems, Foster City, CA, USA). The pBluescript II S/K plasmid containing S-IκB-α cDNA and the expression vector, pcDNA3 (Invitrogen, Leek, The Netherlands) were digested by *Hin*dIII and *Xba*I. Then, the S-IκB-α cDNA was ligated into the *Hin*dIII and *Xba*I sites of pcDNA3 using DNA ligation kit ver. 2 (Takara, Ohtsu, Shiga, Japan). This plasmid was named as pcDNA3-S-IκB. For the Y-IκB plasmid, we used the mutagenic primers for $^{42}$Tyr to $^{42}$Phe, such as MAD-YPU; 5′-GAAAGAC-GAGGAG<u>TTC</u>GAGCAGATGGTCAAGG-3′ and MAD-YPL; 5′-CCTTGACCATCTGCTC<u>GAA</u>CTCCTCGTCTTTC-3′ in the PCR reaction. For the SY-IκB plasmid, the S-IκB-α cDNA was used as a template and the mutagenic primers for $^{42}$Tyr to $^{42}$Phe were used in PCR reaction. The expression plasmids of Y-IκB-α cDNA (pcDNA3-Y-IκB) and SY-IκB-α cDNA (pcDNA3-SY-IκB) were made by similar methods as above-mentioned. A plasmid pcDNA3, without IκB-α cDNA, was used as a negative control.

The methods of transfection were reported previously (Miyakoshi et al, 1997; Yamagishi et al, 1997*a*). Approximately 10$^{6}$ cells of MO54 were transfected with 10 μg of pcDNA3-S-IκB, pcDNA3-Y-IκB, pcDNA3-SY-IκB or pcDNA3 by the electroporation with the unit (model X-CELL 2000, pds Inc., Madison, WI, USA) operated at 800 V. The cells were then cultured in normal medium for 36–48 h, and then cultured in the medium containing G418 (400–800 μg ml$^{-1}$). The cultures were maintained for 2–3 weeks until colonies were formed. The transfection frequencies of these plasmids were 2–8 × 10$^{-4}$.

### Chemicals

Human TNF-α (Pepro Tech EC Ltd., London, UK) was dissolved in distilled water and adriamycin (Wako Pure Chemical Industries, Ltd, Osaka, Japan) was dissolved in medium at a concentration of 20 μg ml$^{-1}$ and 500 μg ml$^{-1}$ respectively. Herbimycin A (Sigma, St Louis, MO, USA) and genistein (Fujikko Ltd, Kobe, Japan) were dissolved in dimethylsulphoxide (DMSO) at a concentration of 2 mg ml$^{-1}$ and 20 mg ml$^{-1}$ respectively. These solutions were diluted in the medium at a final concentration and used for the experiments.

### Western immunoblots and immunoprecipitation

Details of Western immunoblottings were described elsewhere (Miyakoshi et al, 1997). The antibodies used in this experiment were as follows; anti-IκB-α/MAD3 antibody (C-21, Santa Cruz Biotechnology, Inc., Santa Cruz, CA, USA), anti-β-actin antibody (Sigma) and anti-NFκB/p65 antibody (C-20, Santa Cruz Biotechnology). The immunoprecipitation was done by using Protein A Sepharose (Pharmacia Biotechnology). The nuclear extracts were prepared according to Schreiber et al (1989). Autoradiography with enhanced chemiluminescence was done according to the instructions by the manufacturer (Amersham International plc, Buckinghamshire, UK). The densitometric analysis was done by using NIH image 1.60.

### X- and UV-irradiations and cell survival

Methods of X- and UV-irradiations and the analysis of cell survival were described previously (Miyakoshi et al, 1996; Yamagishi et al, 1997*b*). X-irradiation was performed using a Hitachi MBR-1520 at 150 kVp, 20 mA with 0.5 mm Al and 0.1 mm Cu filters with a dose-rate of 0.98–1.02 Gy min$^{-1}$. For UV exposure, a bank of two 15 W Toshiba germicidal UV lamps (predominantly 254 nm) was used with a dose rate of 1.2 J m$^{-2}$ s$^{-1}$. Control plating efficiencies for the parental MO54 cells and the transfected clones are ranged from 0.4 to 0.6. Five replicate plates per experiment were used for each survival point and the experiments were repeated at least three times. Cell survival was statistically analysed by the two-way analysis of variance and by *t*-test for unpaired data.

### Detection of apoptosis

Apoptosis Ladder Detection kit (Wako) was used. The cells were treated with TNF-α (20 ng ml$^{-1}$) for up to 12 h. DNA was extracted and electrophoresed in agarose gel at 100 V. The gel was stained with SYBR Green I (Molecular Probes, Eugene, OR, USA).

### Flow cytometry

Analysis of cell cycle distribution was described previously (Miyakoshi et al, 1997). In brief, cells were suspended in phosphate-buffered saline (PBS) containing 0.1% Triton X-100 after washing twice with PBS. RNAase (1 mg ml$^{-1}$) (Sigma) was added to the cell suspension and the cells were incubated for 30 min at

© 2000 Cancer Research Campaign

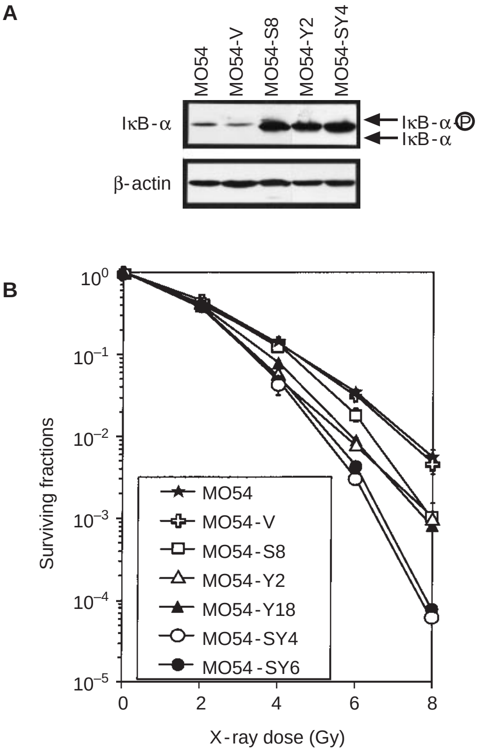

**Figure 1** Expression of S-IκB, Y-IκB and SY-IκB proteins and X-ray sensitivity of the parental cells and the transfected clones. (**A**) The IκB-α protein was detected using anti-IκB-α monoclonal antibody. After removing antibody, the same blot was re-probed with the anti-β-actin monoclonal antibody. (**B**) Exponentially growing cells were exposed to X-rays with various doses. Each point represents the mean of the triplicate experiments with standard deviation

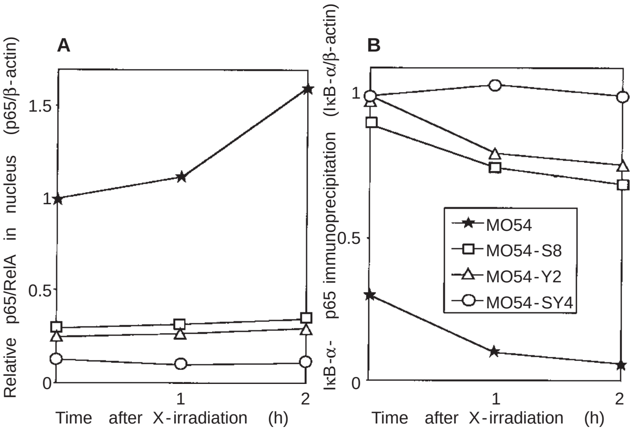

**Figure 2** The relative p65/RelA protein in nuclear fraction and the relative amount of p65/IκB-α complex in MO54-S8, MO54-Y2, MO54-SY4 and the parental MO54 cells after X-irradiation. (**A**) Nuclear extract was prepared from the cells at 1 or 2 h following X-irradiation with 8 Gy. The p65/RelA protein was detected using anti-NFκB/p65 antibody. (**B**) After 1 or 2 h incubation following X-irradiation with 8 Gy, cell lysates were immunoprecipitated with anti-NFκB/p65 antibody and then Western blot with anti-IκB-α monoclonal antibody was done. Each point represents the mean of the duplicate experiments. The densitometric analysis was done by using NIH image 1.60

37°C. Propidium iodide (Molecular Probes) was added at a final concentration of 50 μg ml$^{-1}$ and the cell suspension was left at 4°C for 1 h. The cells were filtered through 50 μm nylon mesh before flow cytometry with a FACScan system (Becton Dickinson Co. Ltd, Franklin Lakes, NJ, USA).

### RESULTS

### X-ray sensitivity of cells expressing S-IκB, Y-IκB and SY-IκB proteins

The expressions of IκB-α in the parental MO54 cells and their S-IκB-, Y-IκB- and SY-IκB-introduced clones are shown in Figure 1A. The clones, MO54-S8, MO54-Y2 and MO54-SY4, were overexpressing S-IκB, Y-IκB and SY-IκB proteins respectively, in which those proteins were non-phosphorylated type. The radiation-dose survival curves of these clones, other expression clones MO54-Y18 and MO54-SY6, the parental MO54 and the negative control of MO54-V are shown in Figure 1B. MO54-S8, MO54-Y2 and MO54-Y18 cells became radiosensitive as compared with the parental MO54 and the vector-transfected MO54-V cells. The cell clones expressing SY-IκB, MO54-SY4 and MO54-SY6, were further radiosensitive. The radiosensitization was estimated from the ratio of mean surviving fraction for the parental cells to that for cells expressing S-IκB, Y-IκB and SY-IκB (Table 1). The calculated additive radiosensitizing value was similar to the ratio of mean surviving fraction of cells expressing SY-IκB. The extent of the radiosensitization in cells expressing SY-IκB was almost additive at higher doses of 4 Gy and over, which means addition of the radiosensitization in each clone expressing S-IκB and Y-IκB. Changes in the amount of p65/RelA protein in nuclear fraction was examined in cells after X-irradiation. The relative amount of p65 protein in nuclear fraction from MO54, MO54-S8, MO54-Y2 and MO54-SY4 cells after X-irradiation is shown in Figure 2A. The constitutive level of p65 in MO54-S8 and MO54-Y2 cells was lower than that in MO54 cells, but this level in MO54-SY4 cells was still lower. After X-irradiation, the amount of p65 in nucleus in MO54 cells was increasing, while that in these clones did not change significantly. The amount of IκB-α/NF-κB complex was also examined after X-irradiation. The relative amount of IκB-α immunoprecipitated with anti-NF-κB/p65 after X-irradiation is shown in Figure 2B. The constitutive level of IκB-α/NF-κB complex observed in MO54-S8, MO54-Y2 and MO54-SY4 cells was higher than that in MO54 cells. After X-irradiation, the amount of the complex in MO54-SY4 cells did not change, but the amount in MO54, MO54-S8 and MO54-Y2 cells was decreased. The IκB-α/NF-κB complex was dissociated and the amount was very low in MO54 cells after X-irradiation.

### Effect of herbimycin A on X-ray sensitivity

We examined whether a phosphorylation inhibitor, herbimycin A, can modify cellular X-ray sensitivity. MO54-S8, MO54-Y2, MO54-Y18, MO54-SY4 and the parental cells were treated with herbimycin A (1 μg ml$^{-1}$) for 2 h, and then irradiated with X-rays. Herbimycin A was removed at 1 h after X-irradiation. Figure 3 shows the X-ray dose–survival curves of these cells. The X-ray-survival of MO54 cells was decreasing by herbimycin A, but did not reach to the radiosensitivity level of those cells expressing SY-IκB protein. The X-ray sensitivity of MO54-S8, MO54-Y2 and MO54-Y18 cells became almost similar level to that of

 © 2000 Cancer Research Campaign

**Table 1** The radiosensitizing value estimated from the ratio of mean surviving fraction at 4, 6 and 8 Gy

| X-ray dose | Ratio of mean surviving fraction [SF(parental cells)/SF(clones)] | | | | |
|---|---|---|---|---|---|
| | Parental cells (MO54) | S-IκB clone (MO54-S8) | Y-IκB clones (MO54-Y2) (MO54-Y18) | SY-IκB clones (MO54-SY4) (MO54-SY6) | Calculated additive radiosensitizing value* |
| 4 Gy | (1.00) | 1.36 | 2.52 | 3.75 | 3.43 |
| 6 Gy | (1.00) | 2.15 | 4.70 | 10.66 | 10.11 |
| 8 Gy | (1.00) | 7.11 | 8.50 | 70.59 | 60.44 |

The radiosensitizing value was estimated from the ratio of mean surviving fraction for the parental cells (MO54) to that for clones expressing S-IκB (MO54-S8), Y-IκB (MO54-Y2 and MO54-Y18) and SY-IκB (MO54-SY4 and MO54-SY6). *The additive radiosensitizing value for SY-IκB was calculated as a multiplication of the surviving fraction ratio for S-IκB and that for Y-IκB.

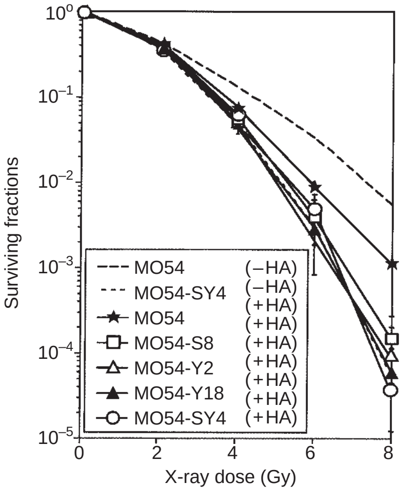

**Figure 3** Effect of herbimycin A on the X-ray sensitivity of the cells expressing S-IκB, Y-IκB and SY-IκB proteins and the parental MO54 cells. Exponentially growing cells were treated with herbimycin A (1 μg ml$^{-1}$) for 2 h and then exposed to X-rays with various doses. Herbimycin A was removed at 1 h after X-irradiation. Each point represents the mean of the triplicate experiments with standard deviation

MO54-SY4 cells. However, the radiosensitivity of MO54-SY4 cells did not change by the treatment with herbimycin A. Treatment with the other inhibitor, genistein (100 μg ml$^{-1}$), showed a similar trend to herbimycin A (data not shown).

### Effect of TNF-α on DNA degradation, cell cycle distribution and X-ray sensitivity

We examined the effect of TNF-α, one of the phosphorylation stimuli, on DNA degradation, cell cycle distribution and X-ray sensitivity. MO54-S8, MO54-Y2, MO54-SY4 and the parental cells were treated with TNF-α (20 ng ml$^{-1}$) for up to 12 h. Figure 4A shows the photographs of DNA gels. TNF-α-induced DNA degradation was observed in MO54-S8, MO54-Y2 and MO54-SY4 cells. The parental MO54 cells did not occur such DNA degradation for up to a 12 h treatment with TNF-α. Patterns of cell cycle distribution after X-irradiation or TNF-α treatment are shown in Figure 4B. Typical G1 arrest and a decrease in S phase ratio were observed in MO54 but not in MO54-SY4 cells after X-irradiation. DNA degradations by the treatment with TNF-α for

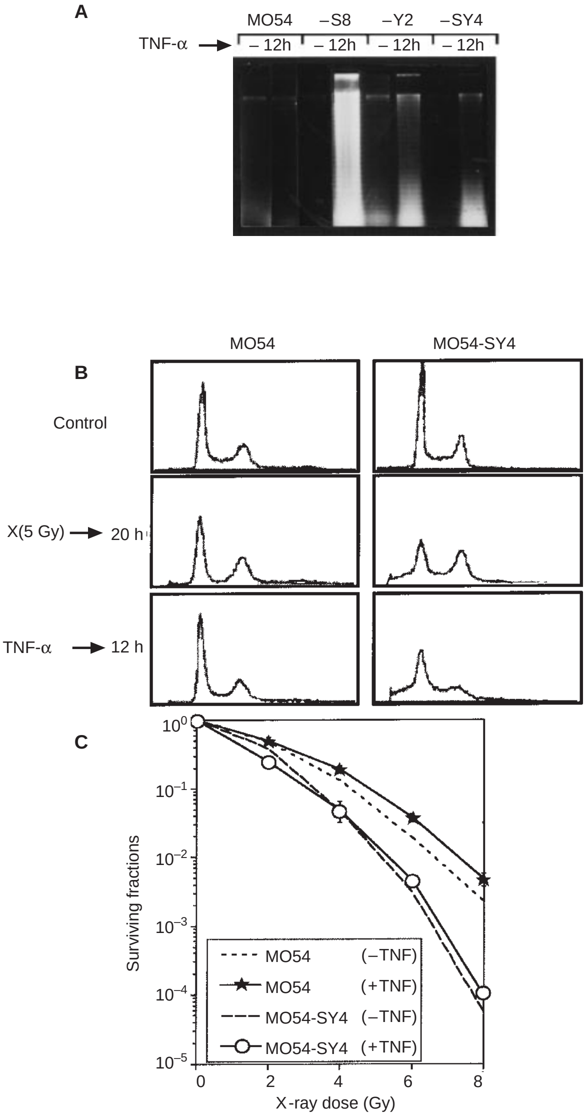

**Figure 4** Ethidium bromide-stained gels, cell cycle distribution and the X-ray sensitivity of cells treated with TNF-α. (**A**) MO54-S8, MO54-Y2, MO54-SY4 and the parental MO54 cells were treated with TNF-α (20 ng ml$^{-1}$) for up to 12 h. DNA from those cells was extracted and electrophoresed. (**B**) Exponentially growing MO54 and MO54-SY4 cells were exposed to X-rays with 5 Gy followed by a 20 h incubation or treated with TNF-α (20 ng ml$^{-1}$) for 12 h, and then cell cycle distribution was analysed by quantitative flow cytometry. Each experiment was repeated twice. (**C**) Exponentially growing cells were treated with TNF-α (20 ng ml$^{-1}$) for 4 h and then exposed to X-rays with various doses. TNF-α was removed at 4 h after X-irradiation. Each point represents the mean of the triplicate experiments with standard deviation

© 2000 Cancer Research Campaign

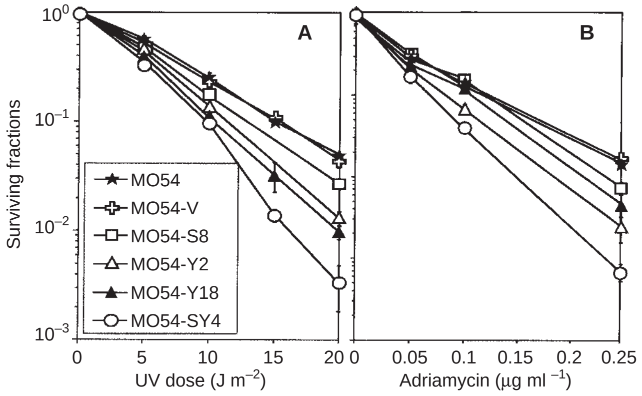

**Figure 5** Adriamycin and UV sensitivities of cells expressing S-IκB, Y-IκB and SY-IκB proteins. Exponentially growing cells were exposed to (**A**) UV with various doses or treated with (**B**) adriamycin for 2 h. Each point represents the mean of the triplicate experiments with standard deviation

12 h were detected in MO54-SY4 but not in the parental MO54 cels.

MO54-SY4 and MO54 cells were treated with TNF-α for 4 h and then irradiated with X-rays. TNF-α was removed at 4 h after X-irradiation. Figure 4C shows the X-ray dose–survival curves of those cells. The toxicity of TNF-α alone, in which the surviving fraction was 0.61 for MO54-SY4, was normalized in the survival curves. No decrease in survival was observed in MO54 cells after treatment with TNF-α. The radiosensitivity of MO54-SY4 cells did not change by the treatment with TNF-α. The X-ray-survival of MO54 cells at doses of 6 and 8 Gy was increasing slightly by the treatment with TNF-α ($P < 0.05$).

### Adriamycin and UV sensitivities

We examined the cellular sensitivity to other DNA damaging agents, adriamycin and UV. Cell survival after adriamycin treatment or UV-irradiation in MO54-S8, MO54-Y2, MO54-Y18, MO54-SY4, MO54-V, and the parental cells are shown in Figure 5A and 5B. MO54-S8, MO54-Y2 and MO54-Y18 cells were sensitive to adriamycin and UV. MO54-SY4 cells were further sensitive to the both, the extent of the sensitization to adriamycin and UV in cells expressing SY-IκB also being additive. Change in the sensitivity to adriamycin and UV in those clones showed a very similar trend to that in the X-ray sensitivity.

### DISCUSSION

For the activation of NF-κB, two different pathways for the phosphorylation of IκB-α are demonstrated, one for serine (at residues 32 and 36) phosphorylation and the other for tyrosine (at residue 42) phosphorylation. Wang et al (1996) reported that inhibition of NF-κB nuclear translocation by the introduction of S-IκB plasmid into human fibrosarcoma cell line HT 1080 enhanced cell killing by ionizing radiation. Our finding using MO54-S8 cells in this study is consistent with their data (Figure 1B). In addition, the clones expressing Y-IκB protein became radiosensitive and the clones expressing SY-IκB protein were further radiosensitive (Figure 1B). The extent of radiosensitization in cells expressing SY-IκB is almost additive (Table 1). Changes in the nuclear fraction of p65/Rel protein and the amount of NF-κB/IκB-α complex after X-irradiation also depended on the status of IκB-α phosphorylation (Figure 2). Therefore, the inhibition of phosphorylation at serine and tyrosine of IκB-α acts independently on the radiosensitization of the cells.

Phosphorylation pathways are important for NF-κB activation. Treatment with a phosphorylation inhibitor, herbimycin A or genistein, inhibits radiation-induced NF-κB activation (Iwasaki et al, 1992; Uckun et al, 1993). The X-ray sensitivity of MO54-SY4 cells did not change by the treatment with herbimycin A, while that of the parental MO54 cells was enhanced by herbimycin A (Figure 3). Genistein had a similar effect of herbimycin A on the X-ray sensitivity of those cells (data not shown). Inhibition of phosphorylations by herbimycin A sensitized cells to X-rays, supports that the radiosensitization of cells expressing S-IκB, Y-IκB and SY-IκB proteins could be, in part, due to the inhibition of IκB-α phosphorylation.

The phosphorylation of IκB-α is induced by a variety of activators, such as TNF-α, 12-*O*-tetradecanoylphorbol-13-acetate (TPA) and interleukin (IL)-1, resulting in the induction of NF-κB activation (Beg et al, 1993; Brown et al, 1993; Cordle et al, 1993; Mellits et al, 1993). The expression of S-IκB protein potentiately enhanced the ability of TNF-α to initiate apoptosis in a variety of cells that are normally resistant to this cytokine, suggests that the activation of NF-κB by TNF-α is protective (Wang et al, 1996). Treatment with TNF-α induced DNA degradation in MO54-S8, MO54-Y2 and MO54-SY4 cells, but not in the parental MO54 cells (Figure 4A). The results of cell cycle distribution of MO54-SY4 and MO54 cells supported these observations (Figure 4B). By the treatment with TNF-α, X-ray survivals of MO54-SY4 cells did not change significantly, but those of MO54 cells increased slightly (Figure 4C). Thus, the absence of NF-κB/p65 sensitized cells to TNF-α, but an increase in cell killing induced by X-irradiation in MO54-SY4 might be independent of the toxic action of TNF-α, such as an induction of DNA degradation. In addition, TNF-α-induced activation of NF-κB in MO54 cells may provide protection against cell killing by X-rays.

For the response of cells to other DNA damaging agents, such as adriamycin and UV, MO54-S8, MO54-Y2, MO54-Y18 and MO54-SY4 cells became sensitive as compared with the parental MO54 cells (Figure 5). In addition, changes in the sensitivity to adriamycin and UV in those clones showed a very similar trend to that in the X-ray sensitivity and the extent of these sensitizations in cells expressing SY-IκB was also additive (Figure 5). Therefore, the sensitization of those cells to adriamycin and UV was also independently induced by the inhibition of serine and tyrosine phosphorylations in IκB-α.

In the treatment of cancer, sensitivity or resistance of tumour cells to ionizing radiation and anti-tumour chemical agents has been substantial clinical consequences. However, molecular mechanisms and/or intrinsic factors controlling these cellular sensitivities are not fully understood in mammalian cells. In the present study, an inactivation of NF-κB by inhibition of both phosphorylations at serine and tyrosine of IκB-α enhances the sensitivity to X-rays and adriamycin in glioma cell line. The present findings suggest that the transcriptional activation induced by NF-κB may play a role in the repair of DNA damages. The present study also proposes a possibility that the inactivation of NF-κB by inhibition of both serine and tyrosine phosphorylations may be useful for the treatment of cancer in radio- and chemotherapies.

© 2000 Cancer Research Campaign

### ACKNOWLEDGEMENTS

We are grateful to A Yamada, M Yoshida and H Yaguchi for excellent technical assistant in tissue culture. The cooperations of Drs J Inoue and RS Day for supplies of IκB-α cDNA and human malignant glioma cell lines, respectively, are gratefully acknowledged. This work was supported in part by a Grant-in-Aid for Scientific Research on Priority Areas (09255227 and 10153232) from the Ministry of Education, Science, Sports and Culture, Japan.

### REFERENCES

Alkalay I, Yaron A, Hatzubai A, Orian A, Ciechanover A and Ben-Neriah Y (1995) Stimulation-dependent IκBα phosphorylation marks the NF-κB inhibitor for degradation via the ubiquitin-proteasome pathway. *Proc Natl Acad Sci USA* **92**: 10599–10603

Baeuerle PA (1991) The inducible transcription activator NF-κB: regulation by distinct protein subunits. *Biochim Biophys Acta* **1072**: 63–80

Baeuerle PA and Baltimore D (1988) IκB-α: a specific inhibitor of the NF-κB transcription factor. *Science* **242**: 540–546

Baeuerle PA and Henkel T (1994) Function and activation of NF-κB in immune system. *Annu Rev Immunol* **12**: 141–179

Beg AA, Rubin SM, Scheinman RI, Haskill S, Rosen CA and Baldwin AS (1992) IκB interacts with the nuclear locatization sequencers of the subunits of NF-κB: a mechanism for cytoplasmic retention. *Genes Dev* **6**: 1899–1913

Beg AA, Finc TS, Nantermet PV and Baldwin AS Jr (1993) Tumor necrosis factor and interleukin-1 lead to phosphorylation and loss of IκB-α: a mechanism for NF-κB activation. *Mol Cell Biol* **13**: 13301–13310

Brockman JA, Scherer DC, McKinsey TA, Hall SM, Qi X, Young Lee W and Ballard DW (1995) Coupling of a signal response domain in IκBα to multiple pathways for NF-κB activation. *Mol Cell Biol* **15**: 2809–2818

Brown K, Park S, Kanno T, Franzoso G and Siebenlist U (1993) Mutual regulation of the transcriptional activator NF-κB and its inhibitor, I kappa B-α. *Proc Natl Acad Sci USA* **90**: 2532–2536

Brown K, Gerstberger S, Carlson L, Franzoso G and Siebenlist U (1995) Control of IκB-α proteolysis by site-specific, signal-induced phosphorylation. *Science* **267**: 1485–1488

Cordle SR, Donald R, Read MA and Hawiger J (1993) Lipopolysaccharide induces phosphorylation of MAD3 and activation of c-Rel and related NF-κB proteins in human monocytic THP-1 cells. *J Biol Chem* **268**: 11803–11810

Hallahan DE, Sukhatme VP, Sherman ML, Virudaghalam S, Kufe D and Weichselbaum RR (1991) Protein kinase C mediates x-ray inducibility of nuclear signal transducers EGR1 and JUN. *Proc Natl Acad Sci USA* **88**: 2156–2160

Imbert V, Rupec RA, Livolsi A, Pah HL, Traenckner EB-M, Mueller-Dieckman C, Farahifer D, Rossi B, Auberger P, Baeuerle PA and Peyron J-F (1996) Tyrosine phosphorylation of IκB-α activates NF-κB without proteolytic degradation of IκB-α. *Cell* **86**: 787–798

Iwasaki T, Uehara Y, Gravea L, Rachie N and Bomsztyk K (1992) Herbimycin A blocks IL-1-induced NF-κB DNA-binding activity in lymphoid cell lines. *FEBS Lett* **298**: 240–244

Jung M, Zhang Y, Lee S and Dritschilo A (1995) Correction of radiation sensitivity in Ataxia Telangiectasia cells by a truncated IκB-α. *Science* **268**: 1619–1621

Kasid U, Pfeifer A, Brenman T, Beckett M, Weichselbaum RR, Dritschilo A and Mark GE (1989) Effect of antisense c-*raf*-1 on tumorigenicity and radiation sensitivity of a human squamous carcinoma. *Science* **243**: 1354–1356

Mellits KH, Hay RT and Goodbourn S (1993) Proteolytic degradation of MAD3 (IκB alpha) and enhanced processing of the NF-κB precursor p105 are obligatory steps in the activation of NF-κB. *Nucelic Acids Res* **21**: 5059–5066

Miyakoshi J, Yamagishi N, Ohtsu S and Takebe H (1995) Changes in radiation sensitivity of human osteosarcoma cells after p53 introduction. *Jap J Cancer Res* **86**: 711–713

Miyakoshi J, Yamagishi N, Ohtsu S, Mohri K and Takebe H (1996) Exposure to magnetic field (5 mT at 60 Hz) does not affect cell growth and c-myc gene expression. *Mutat Res* **349**: 109–114

Miyakoshi J, Kitagawa K, Yamagishi N, Ohtsu S, Day RS III and Takebe H (1997) Increased radiosensitivity of p16 gene-deleted human glioma cells after transfection with wild-type p16 gene. *Jap J Cancer Res* **88**: 34–38

Morhan N and Meltz ML (1994) Induction of nuclear factor κB after low-dose ionizing radiation involves a reactive oxygen intermediate signaling pathway. *Radiat Res* **140**: 97–104

Pirollo KF, Tong YA, Villegas Z, Chen Y and Chang EH (1993) Oncogene-transformed NIH 3T3 cells display radiation resistance levels indicative of a signal transduction pathway leading to the radiation-resistant phenotype. *Radiat Res* **135**: 234–243

Scherer DC, Brockman JA, Chen Z, Maniatis T and Ballard DW (1995) Signal-induced degradation of IκBα requires site-specific ubiquitination. *Proc Natl Acad Sci USA* **92**: 11259–11263

Schreiber E, Matthias P, Mulle MM and Schaffner W (1989) Rapid detection of octamer binding proteins with 'mini-extracts', prepared from a small number of cells. *Nucleic Acids Res* **17**: 6419

Sklar MD (1988) The ras oncogenes increase the intrinsic resistance of HIH 3T3 cells to ionizing radiation. *Science* **239**: 645–647

Traenckner EB-M, Pahl HL, Henke T, Schmidt KN, Wilk S and Baererle PA (1995) Phosphorylation of human IκB-α on serines 32 and 36 control IκB-α proteolysis and NF-κB activation in response to diverse stimuli. *EMBO J* **14**: 2876–2883

Uckun FM, Schieven GL, Tuel-Ahlgren LM, Dibirdik I, Myers DE, Ledbetter JA and Song CW (1993) Tyrosine phosphorylation is a mandatory proximal step in radiation-induced activation of the protein kinase C signaling pathway in human B-lymphocyte precursors. *Proc Natl Acad Sci USA* **90**: 252–256

Yamagishi N, Miyakoshi J and Takebe H (1997*a*) Decrease in the frequency of x-ray-induced mutation by wild-type p53 protein in human osteosarcoma cells. *Carcinogenesis* **18**: 695–700

Yamagishi N, Miyakoshi J, Yagi T and Takebe H (1997*b*) Suppression of UV-induced mutation by wild-type p53 protein in human osteosarcoma cells. *Mutagenesis* **12**: 191–194

Verma IM, Stevenson JK, Schwarz EM, Van Antwerp D and Miyamoto S (1995) Rel/NF-κB family: intimate tales of association and dissociation. *Genes Dev* **9**: 2723–2735

Wang C-Y, Mayo MW and Baldwin AS Jr (1996) TNF- and cancer therapy-induced apoptosis: potentiation by inhibition of NF-κB. *Science* **274**: 784–787

Wilson RE, Taylor SL, Atherton GT, Johnston D, Waters CM and Norton JD (1993) Early response gene signalling cascades activated by ionizing radiation in primary human B cells. *Oncogene* **8**: 3229–3237

© 2000 Cancer Research Campaign 
